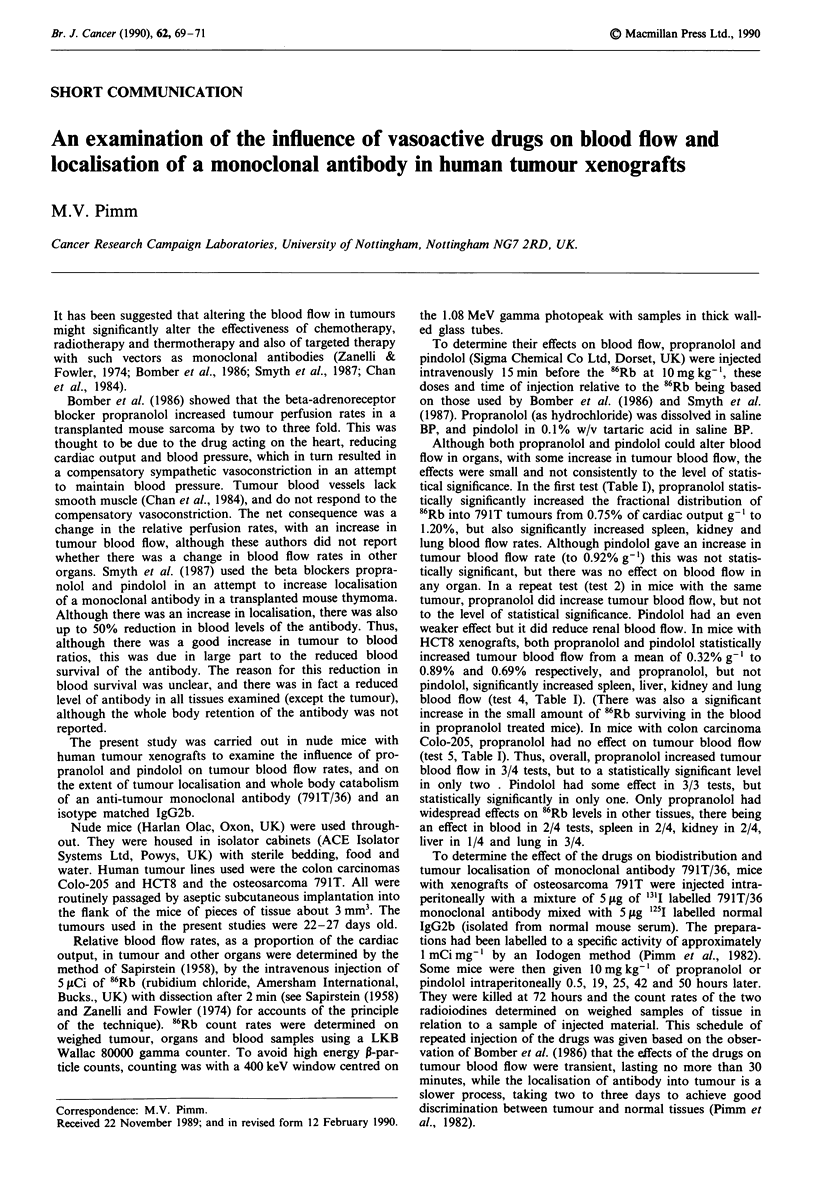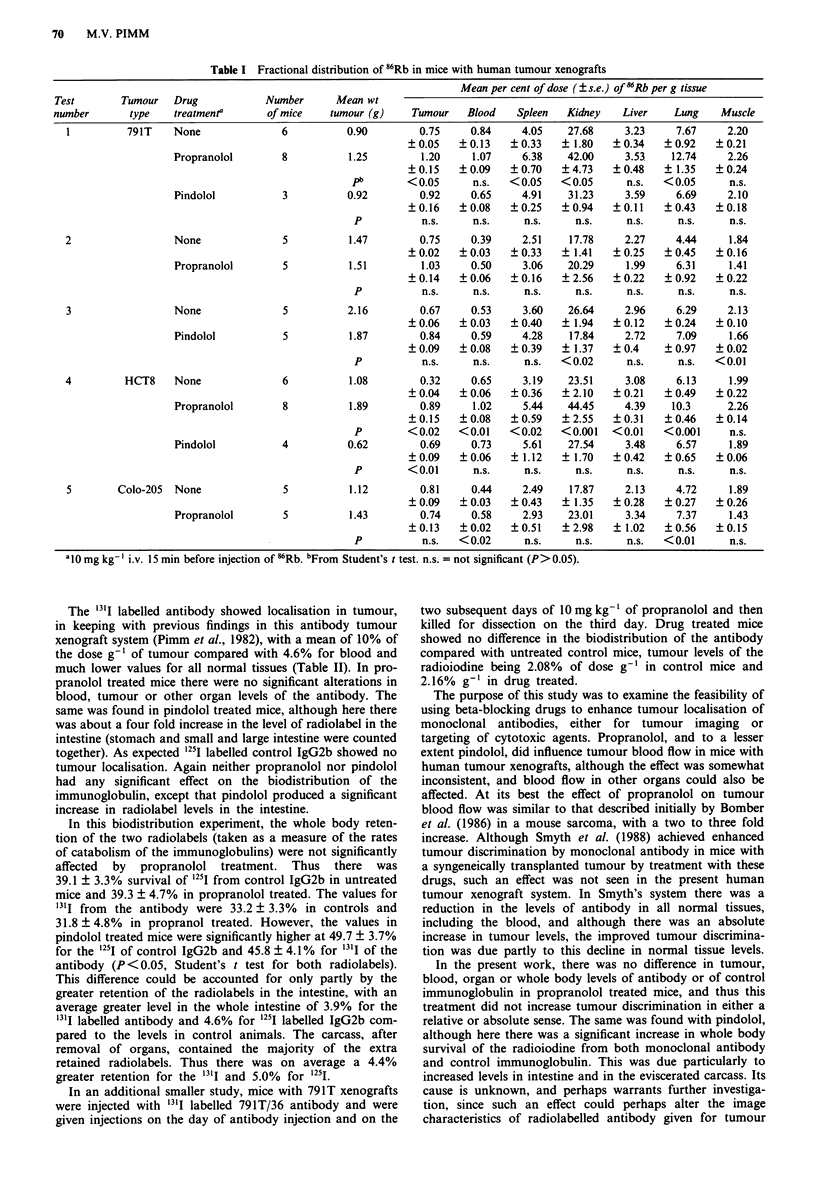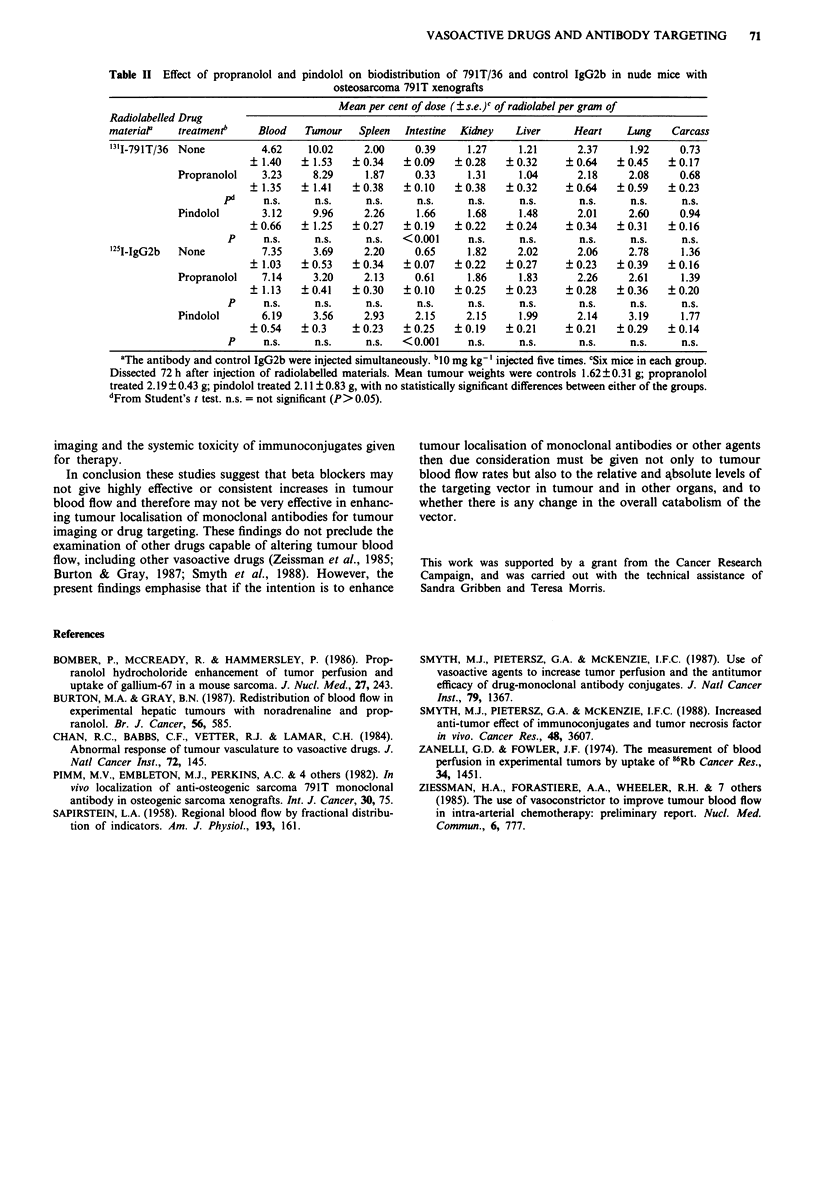# An examination of the influence of vasoactive drugs on blood flow and localisation of a monoclonal antibody in human tumour xenografts.

**DOI:** 10.1038/bjc.1990.231

**Published:** 1990-07

**Authors:** M. V. Pimm

**Affiliations:** Cancer Research Campaign Laboratories, University of Nottingham, UK.


Br. J. Cancer (1990), 62, 69–71 © Macmillan Press Ltd., 1990

SHORT COMMUNICATION

### An examination of the influence of vasoactive drugs on blood flow and localisation of a monoclonal antibody in human tumour xenografts

M.V. Pimm

*Cancer Research Campaign Laboratories, University of Nottingham, Nottingham NG7 2RD, UK.*

It has been suggested that altering the blood flow in tumours might significantly alter the effectiveness of chemotherapy, radiotherapy and thermotherapy and also of targeted therapy with such vectors as monoclonal antibodies (Zanelli & Fowler, 1974; Bomber *et al.*, 1986; Smyth *et al.*, 1987; Chan *et al.*, 1984).

Bomber *et al.* (1986) showed that the beta-adrenoreceptor blocker propranolol increased tumour perfusion rates in a transplanted mouse sarcoma by two to three fold. This was thought to be due to the drug acting on the heart, reducing cardiac output and blood pressure, which in turn resulted in a compensatory sympathetic vasoconstriction in an attempt to maintain blood pressure. Tumour blood vessels lack smooth muscle (Chan *et al.*, 1984), and do not respond to the compensatory vasoconstriction. The net consequence was a change in the relative perfusion rates, with an increase in tumour blood flow, although these authors did not report whether there was a change in blood flow rates in other organs. Smyth *et al.* (1987) used the beta blockers propranolol and pindolol in an attempt to increase localisation of a monoclonal antibody in a transplanted mouse thymoma. Although there was an increase in localisation, there was also up to 50% reduction in blood levels of the antibody. Thus, although there was a good increase in tumour to blood ratios, this was due in large part to the reduced blood survival of the antibody. The reason for this reduction in blood survival was unclear, and there was in fact a reduced level of antibody in all tissues examined (except the tumour), although the whole body retention of the antibody was not reported.

The present study was carried out in nude mice with human tumour xenografts to examine the influence of propranolol and pindolol on tumour blood flow rates, and on the extent of tumour localisation and whole body catabolism of an anti-tumour monoclonal antibody (791T/36) and an isotype matched IgG2b.

Nude mice (Harlan Olac, Oxon, UK) were used throughout. They were housed in isolator cabinets (ACE Isolator Systems Ltd, Powys, UK) with sterile bedding, food and water. Human tumour lines used were the colon carcinomas Colo-205 and HCT8 and the osteosarcoma 791T. All were routinely passaged by aseptic subcutaneous implantation into the flank of the mice of pieces of tissue about 3 $mm^3$. The tumours used in the present studies were 22–27 days old.

Relative blood flow rates, as a proportion of the cardiac output, in tumour and other organs were determined by the method of Sapirstein (1958), by the intravenous injection of 5 $\mu$Ci of $^{86}$Rb (rubidium chloride, Amersham International, Bucks., UK) with dissection after 2 min (see Sapirstein (1958) and Zanelli and Fowler (1974) for accounts of the principle of the technique). $^{86}$Rb count rates were determined on weighed tumour, organs and blood samples using a LKB Wallac 80000 gamma counter. To avoid high energy $\beta$-particle counts, counting was with a 400 keV window centred on the 1.08 MeV gamma photopeak with samples in thick walled glass tubes.

To determine their effects on blood flow, propranolol and pindolol (Sigma Chemical Co Ltd, Dorset, UK) were injected intravenously 15 min before the $^{86}$Rb at 10 mg $kg^{-1}$, these doses and time of injection relative to the $^{86}$Rb being based on those used by Bomber *et al.* (1986) and Smyth *et al.* (1987). Propranolol (as hydrochloride) was dissolved in saline BP, and pindolol in 0.1% w/v tartaric acid in saline BP.

Although both propranolol and pindolol could alter blood flow in organs, with some increase in tumour blood flow, the effects were small and not consistently to the level of statistical significance. In the first test (Table I), propranolol statistically significantly increased the fractional distribution of $^{86}$Rb into 791T tumours from 0.75% of cardiac output $g^{-1}$ to 1.20%, but also significantly increased spleen, kidney and lung blood flow rates. Although pindolol gave an increase in tumour blood flow rate (to 0.92% $g^{-1}$) this was not statistically significant, but there was no effect on blood flow in any organ. In a repeat test (test 2) in mice with the same tumour, propranolol did increase tumour blood flow, but not to the level of statistical significance. Pindolol had an even weaker effect but it did reduce renal blood flow. In mice with HCT8 xenografts, both propranolol and pindolol statistically increased tumour blood flow from a mean of 0.32% $g^{-1}$ to 0.89% and 0.69% respectively, and propranolol, but not pindolol, significantly increased spleen, liver, kidney and lung blood flow (test 4, Table I). (There was also a significant increase in the small amount of $^{86}$Rb surviving in the blood in propranolol treated mice). In mice with colon carcinoma Colo-205, propranolol had no effect on tumour blood flow (test 5, Table I). Thus, overall, propranolol increased tumour blood flow in 3/4 tests, but to a statistically significant level in only two . Pindolol had some effect in 3/3 tests, but statistically significantly in only one. Only propranolol had widespread effects on $^{86}$Rb levels in other tissues, there being an effect in blood in 2/4 tests, spleen in 2/4, kidney in 2/4, liver in 1/4 and lung in 3/4.

To determine the effect of the drugs on biodistribution and tumour localisation of monoclonal antibody 791T/36, mice with xenografts of osteosarcoma 791T were injected intraperitoneally with a mixture of 5 $\mu$g of $^{131}$I labelled 791T/36 monoclonal antibody mixed with 5 $\mu$g $^{125}$I labelled normal IgG2b (isolated from normal mouse serum). The preparations had been labelled to a specific activity of approximately 1 mCi $mg^{-1}$ by an Iodogen method (Pimm *et al.*, 1982). Some mice were then given 10 mg $kg^{-1}$ of propranolol or pindolol intraperitoneally 0.5, 19, 25, 42 and 50 hours later. They were killed at 72 hours and the count rates of the two radioiodines determined on weighed samples of tissue in relation to a sample of injected material. This schedule of repeated injection of the drugs was given based on the observation of Bomber *et al.* (1986) that the effects of the drugs on tumour blood flow were transient, lasting no more than 30 minutes, while the localisation of antibody into tumour is a slower process, taking two to three days to achieve good discrimination between tumour and normal tissues (Pimm *et al.*, 1982).

Correspondence: M.V. Pimm.
Received 22 November 1989; and in revised form 12 February 1990.

**Table I** Fractional distribution of $^{86}$Rb in mice with human tumour xenografts

| Test number | Tumour type | Drug treatment[a] | Number of mice | Mean wt tumour (g) | *Mean per cent of dose (±s.e.) of $^{86}$Rb per g tissue* Tumour | Blood | Spleen | Kidney | Liver | Lung | Muscle |
|---|---|---|---|---|---|---|---|---|---|---|---|
| 1 | 791T | None | 6 | 0.90 | 0.75 | 0.84 | 4.05 | 27.68 | 3.23 | 7.67 | 2.20 |
| | | | | | ±0.05 | ±0.13 | ±0.33 | ±1.80 | ±0.34 | ±0.92 | ±0.21 |
| | | Propranolol | 8 | 1.25 | 1.20 | 1.07 | 6.38 | 42.00 | 3.53 | 12.74 | 2.26 |
| | | | | | ±0.15 | ±0.09 | ±0.70 | ±4.73 | ±0.48 | ±1.35 | ±0.24 |
| | | | | *P*[b] | <0.05 | n.s. | <0.05 | <0.05 | n.s. | <0.05 | n.s. |
| | | Pindolol | 3 | 0.92 | 0.92 | 0.65 | 4.91 | 31.23 | 3.59 | 6.69 | 2.10 |
| | | | | | ±0.16 | ±0.08 | ±0.25 | ±0.94 | ±0.11 | ±0.43 | ±0.18 |
| | | | | *P* | n.s. | n.s. | n.s. | n.s. | n.s. | n.s. | n.s. |
| 2 | | None | 5 | 1.47 | 0.75 | 0.39 | 2.51 | 17.78 | 2.27 | 4.44 | 1.84 |
| | | | | | ±0.02 | ±0.03 | ±0.33 | ±1.41 | ±0.25 | ±0.45 | ±0.16 |
| | | Propranolol | 5 | 1.51 | 1.03 | 0.50 | 3.06 | 20.29 | 1.99 | 6.31 | 1.41 |
| | | | | | ±0.14 | ±0.06 | ±0.16 | ±2.56 | ±0.22 | ±0.92 | ±0.22 |
| | | | | *P* | n.s. | n.s. | n.s. | n.s. | n.s. | n.s. | n.s. |
| 3 | | None | 5 | 2.16 | 0.67 | 0.53 | 3.60 | 26.64 | 2.96 | 6.29 | 2.13 |
| | | | | | ±0.06 | ±0.03 | ±0.40 | ±1.94 | ±0.12 | ±0.24 | ±0.10 |
| | | Pindolol | 5 | 1.87 | 0.84 | 0.59 | 4.28 | 17.84 | 2.72 | 7.09 | 1.66 |
| | | | | | ±0.09 | ±0.08 | ±0.39 | ±1.37 | ±0.4 | ±0.97 | ±0.02 |
| | | | | *P* | n.s. | n.s. | n.s. | <0.02 | n.s. | n.s. | <0.01 |
| 4 | HCT8 | None | 6 | 1.08 | 0.32 | 0.65 | 3.19 | 23.51 | 3.08 | 6.13 | 1.99 |
| | | | | | ±0.04 | ±0.06 | ±0.36 | ±2.10 | ±0.21 | ±0.49 | ±0.22 |
| | | Propranolol | 8 | 1.89 | 0.89 | 1.02 | 5.44 | 44.45 | 4.39 | 10.3 | 2.26 |
| | | | | | ±0.15 | ±0.08 | ±0.59 | ±2.55 | ±0.31 | ±0.46 | ±0.14 |
| | | | | *P* | <0.02 | <0.01 | <0.02 | <0.001 | <0.01 | <0.001 | n.s. |
| | | Pindolol | 4 | 0.62 | 0.69 | 0.73 | 5.61 | 27.54 | 3.48 | 6.57 | 1.89 |
| | | | | | ±0.09 | ±0.06 | ±1.12 | ±1.70 | ±0.42 | ±0.65 | ±0.06 |
| | | | | *P* | <0.01 | n.s. | n.s. | n.s. | n.s. | n.s. | n.s. |
| 5 | Colo-205 | None | 5 | 1.12 | 0.81 | 0.44 | 2.49 | 17.87 | 2.13 | 4.72 | 1.89 |
| | | | | | ±0.09 | ±0.03 | ±0.43 | ±1.35 | ±0.28 | ±0.27 | ±0.26 |
| | | Propranolol | 5 | 1.43 | 0.74 | 0.58 | 2.93 | 23.01 | 3.34 | 7.37 | 1.43 |
| | | | | | ±0.13 | ±0.02 | ±0.51 | ±2.98 | ±1.02 | ±0.56 | ±0.15 |
| | | | | *P* | n.s. | <0.02 | n.s. | n.s. | n.s. | <0.01 | n.s. |

[a]10 mg kg$^{-1}$ i.v. 15 min before injection of $^{86}$Rb. [b]From Student's *t* test. n.s. = not significant ($P > 0.05$).

The $^{131}$I labelled antibody showed localisation in tumour, in keeping with previous findings in this antibody tumour xenograft system (Pimm *et al.*, 1982), with a mean of 10% of the dose g$^{-1}$ of tumour compared with 4.6% for blood and much lower values for all normal tissues (Table II). In propranolol treated mice there were no significant alterations in blood, tumour or other organ levels of the antibody. The same was found in pindolol treated mice, although here there was about a four fold increase in the level of radiolabel in the intestine (stomach and small and large intestine were counted together). As expected $^{125}$I labelled control IgG2b showed no tumour localisation. Again neither propranolol nor pindolol had any significant effect on the biodistribution of the immunoglobulin, except that pindolol produced a significant increase in radiolabel levels in the intestine.

In this biodistribution experiment, the whole body retention of the two radiolabels (taken as a measure of the rates of catabolism of the immunoglobulins) were not significantly affected by propranolol treatment. Thus there was 39.1 ± 3.3% survival of $^{125}$I from control IgG2b in untreated mice and 39.3 ± 4.7% in propranolol treated. The values for $^{131}$I from the antibody were 33.2 ± 3.3% in controls and 31.8 ± 4.8% in propranol treated. However, the values in pindolol treated mice were significantly higher at 49.7 ± 3.7% for the $^{125}$I of control IgG2b and 45.8 ± 4.1% for $^{131}$I of the antibody ($P < 0.05$, Student's *t* test for both radiolabels). This difference could be accounted for only partly by the greater retention of the radiolabels in the intestine, with an average greater level in the whole intestine of 3.9% for the $^{131}$I labelled antibody and 4.6% for $^{125}$I labelled IgG2b compared to the levels in control animals. The carcass, after removal of organs, contained the majority of the extra retained radiolabels. Thus there was on average a 4.4% greater retention for the $^{131}$I and 5.0% for $^{125}$I.

In an additional smaller study, mice with 791T xenografts were injected with $^{131}$I labelled 791T/36 antibody and were given injections on the day of antibody injection and on the two subsequent days of 10 mg kg$^{-1}$ of propranolol and then killed for dissection on the third day. Drug treated mice showed no difference in the biodistribution of the antibody compared with untreated control mice, tumour levels of the radioiodine being 2.08% of dose g$^{-1}$ in control mice and 2.16% g$^{-1}$ in drug treated.

The purpose of this study was to examine the feasibility of using beta-blocking drugs to enhance tumour localisation of monoclonal antibodies, either for tumour imaging or targeting of cytotoxic agents. Propranolol, and to a lesser extent pindolol, did influence tumour blood flow in mice with human tumour xenografts, although the effect was somewhat inconsistent, and blood flow in other organs could also be affected. At its best the effect of propranolol on tumour blood flow was similar to that described initially by Bomber *et al.* (1986) in a mouse sarcoma, with a two to three fold increase. Although Smyth *et al.* (1988) achieved enhanced tumour discrimination by monoclonal antibody in mice with a syngeneically transplanted tumour by treatment with these drugs, such an effect was not seen in the present human tumour xenograft system. In Smyth's system there was a reduction in the levels of antibody in all normal tissues, including the blood, and although there was an absolute increase in tumour levels, the improved tumour discrimination was due partly to this decline in normal tissue levels.

In the present work, there was no difference in tumour, blood, organ or whole body levels of antibody or of control immunoglobulin in propranolol treated mice, and thus this treatment did not increase tumour discrimination in either a relative or absolute sense. The same was found with pindolol, although here there was a significant increase in whole body survival of the radioiodine from both monoclonal antibody and control immunoglobulin. This was due particularly to increased levels in intestine and in the eviscerated carcass. Its cause is unknown, and perhaps warrants further investigation, since such an effect could perhaps alter the image characteristics of radiolabelled antibody given for tumour

**Table II** Effect of propranolol and pindolol on biodistribution of 791T/36 and control IgG2b in nude mice with osteosarcoma 791T xenografts

| Radiolabelled material[a] | Drug treatment[b] | Mean per cent of dose (±s.e.)[c] of radiolabel per gram of: Blood | Tumour | Spleen | Intestine | Kidney | Liver | Heart | Lung | Carcass |
|---|---|---|---|---|---|---|---|---|---|---|
| $^{131}$I-791T/36 | None | 4.62 | 10.02 | 2.00 | 0.39 | 1.27 | 1.21 | 2.37 | 1.92 | 0.73 |
| | | ±1.40 | ±1.53 | ±0.34 | ±0.09 | ±0.28 | ±0.32 | ±0.64 | ±0.45 | ±0.17 |
| | Propranolol | 3.23 | 8.29 | 1.87 | 0.33 | 1.31 | 1.04 | 2.18 | 2.08 | 0.68 |
| | | ±1.35 | ±1.41 | ±0.38 | ±0.10 | ±0.38 | ±0.32 | ±0.64 | ±0.59 | ±0.23 |
| | $P^d$ | n.s. | n.s. | n.s. | n.s. | n.s. | n.s. | n.s. | n.s. | n.s. |
| | Pindolol | 3.12 | 9.96 | 2.26 | 1.66 | 1.68 | 1.48 | 2.01 | 2.60 | 0.94 |
| | | ±0.66 | ±1.25 | ±0.27 | ±0.19 | ±0.22 | ±0.24 | ±0.34 | ±0.31 | ±0.16 |
| | $P$ | n.s. | n.s. | n.s. | <0.001 | n.s. | n.s. | n.s. | n.s. | n.s. |
| $^{125}$I-IgG2b | None | 7.35 | 3.69 | 2.20 | 0.65 | 1.82 | 2.02 | 2.06 | 2.78 | 1.36 |
| | | ±1.03 | ±0.53 | ±0.34 | ±0.07 | ±0.22 | ±0.27 | ±0.23 | ±0.39 | ±0.16 |
| | Propranolol | 7.14 | 3.20 | 2.13 | 0.61 | 1.86 | 1.83 | 2.26 | 2.61 | 1.39 |
| | | ±1.13 | ±0.41 | ±0.30 | ±0.10 | ±0.25 | ±0.23 | ±0.28 | ±0.36 | ±0.20 |
| | $P$ | n.s. | n.s. | n.s. | n.s. | n.s. | n.s. | n.s. | n.s. | n.s. |
| | Pindolol | 6.19 | 3.56 | 2.93 | 2.15 | 2.15 | 1.99 | 2.14 | 3.19 | 1.77 |
| | | ±0.54 | ±0.3 | ±0.23 | ±0.25 | ±0.19 | ±0.21 | ±0.21 | ±0.29 | ±0.14 |
| | $P$ | n.s. | n.s. | n.s. | <0.001 | n.s. | n.s. | n.s. | n.s. | n.s. |

[a]The antibody and control IgG2b were injected simultaneously. [b]10 mg kg$^{-1}$ injected five times. [c]Six mice in each group. Dissected 72 h after injection of radiolabelled materials. Mean tumour weights were controls 1.62±0.31 g; propranolol treated 2.19±0.43 g; pindolol treated 2.11±0.83 g, with no statistically significant differences between either of the groups. [d]From Student's $t$ test. n.s. = not significant ($P>0.05$).

imaging and the systemic toxicity of immunoconjugates given for therapy.

In conclusion these studies suggest that beta blockers may not give highly effective or consistent increases in tumour blood flow and therefore may not be very effective in enhancing tumour localisation of monoclonal antibodies for tumour imaging or drug targeting. These findings do not preclude the examination of other drugs capable of altering tumour blood flow, including other vasoactive drugs (Zeissman *et al.*, 1985; Burton & Gray, 1987; Smyth *et al.*, 1988). However, the present findings emphasise that if the intention is to enhance tumour localisation of monoclonal antibodies or other agents then due consideration must be given not only to tumour blood flow rates but also to the relative and absolute levels of the targeting vector in tumour and in other organs, and to whether there is any change in the overall catabolism of the vector.

This work was supported by a grant from the Cancer Research Campaign, and was carried out with the technical assistance of Sandra Gribben and Teresa Morris.

### References

BOMBER, P., MCCREADY, R. & HAMMERSLEY, P. (1986). Propranolol hydrocholoride enhancement of tumor perfusion and uptake of gallium-67 in a mouse sarcoma. *J. Nucl. Med.*, **27**, 243.

BURTON, M.A. & GRAY, B.N. (1987). Redistribution of blood flow in experimental hepatic tumours with noradrenaline and propranolol. *Br. J. Cancer*, **56**, 585.

CHAN, R.C., BABBS, C.F., VETTER, R.J. & LAMAR, C.H. (1984). Abnormal response of tumour vasculature to vasoactive drugs. *J. Natl Cancer Inst.*, **72**, 145.

PIMM, M.V., EMBLETON, M.J., PERKINS, A.C. & 4 others (1982). *In vivo* localization of anti-osteogenic sarcoma 791T monoclonal antibody in osteogenic sarcoma xenografts. *Int. J. Cancer*, **30**, 75.

SAPIRSTEIN, L.A. (1958). Regional blood flow by fractional distribution of indicators. *Am. J. Physiol.*, **193**, 161.

SMYTH, M.J., PIETERSZ, G.A. & MCKENZIE, I.F.C. (1987). Use of vasoactive agents to increase tumor perfusion and the antitumor efficacy of drug-monoclonal antibody conjugates. *J. Natl Cancer Inst.*, **79**, 1367.

SMYTH, M.J., PIETERSZ, G.A. & MCKENZIE, I.F.C. (1988). Increased anti-tumor effect of immunoconjugates and tumor necrosis factor *in vivo*. *Cancer Res.*, **48**, 3607.

ZANELLI, G.D. & FOWLER, J.F. (1974). The measurement of blood perfusion in experimental tumors by uptake of $^{86}$Rb *Cancer Res.*, **34**, 1451.

ZIESSMAN, H.A., FORASTIERE, A.A., WHEELER, R.H. & 7 others (1985). The use of vasoconstrictor to improve tumour blood flow in intra-arterial chemotherapy: preliminary report. *Nucl. Med. Commun.*, **6**, 777.